# High-Frequency Ultrasound-Guided Treatment of a Head and Neck Lymphatic Malformation

**DOI:** 10.3390/healthcare14121717

**Published:** 2026-06-15

**Authors:** Fausto Fiori, Donato Setola, Antonio Romano, Ciro Emiliano Boschetti, Beatriz Nascimento Figueiredo Lebre Martins, Alberta Lucchese, Dario Di Stasio

**Affiliations:** 1 Multidisciplinary Department of Medical-Surgical and Dental Specialties, University of Campania “Luigi Vanvitelli”, 80138 Naples, Italy; fausto.fiori@outlook.com (F.F.); donato.setola@unicampania.it (D.S.); c.e.boschetti@unilink.it (C.E.B.); alberta.lucchese@unicampania.it (A.L.); dario.distasio@unicampania.it (D.D.S.); 2 Department of Oral Diagnosis, Piracicaba Dental School, University of Campinas (FOP-UNICAMP), Piracicaba 13414-903, Brazil

**Keywords:** lymphatic malformation, vascular anomalies, head and neck, doxycycline, sclerotherapy, ultrasound-guided therapy, high-frequency ultrasound, case report

## Abstract

Lymphatic malformations (LMs) are rare congenital low-flow vascular anomalies that frequently involve the head and neck and may be managed with surgery, laser therapy, sclerotherapy, or multimodal approaches depending on lesion type, size, depth, and relationship with adjacent structures. Ultrasound-guided sclerotherapy with doxycycline is an established treatment option for macrocystic lesions, whereas the practical role of high-frequency superficial ultrasound as a technical adjunct has been less specifically discussed. We report the case of a 32-year-old man presenting with a painless left submandibular swelling of approximately two years’ duration. Magnetic resonance imaging showed a well-encapsulated cystic lesion measuring 56 × 35 mm in the left submandibular region, extending into the internal paralaryngeal space and causing mild compression of the laryngeal wall. Previous fine-needle aspiration cytology had not conclusively established the lymphatic nature of the lesion; therefore, an incisional biopsy was performed and confirmed a macrocystic LM. The patient underwent day-surgery intralesional doxycycline sclerotherapy under real-time high-frequency ultrasound guidance using an 18 MHz hockey-stick transducer. After aspiration of the main cystic compartment through a 25-gauge needle, 100 mg of doxycycline diluted to 10 mg/mL in normal saline was slowly injected under continuous visualization. The procedure was well tolerated under topical local anesthesia, without pain, complications, or adverse effects. A partial clinical reduction was observed after the first session; the treatment was repeated after three months, resulting in apparent complete clinical resolution at one-year follow-up; no post-treatment imaging was available to confirm radiological resolution. This case highlights the potential technical value of high-frequency superficial ultrasonography, particularly for needle positioning, improved delineation of superficial locules, and real-time monitoring of sclerosant distribution.

## 1. Introduction

Lymphatic malformations are congenital low-flow vascular anomalies currently classified within the International Society for the Study of Vascular Anomalies (ISSVA) framework as lymphatic anomalies [1,2]. They may be subdivided into macrocystic, microcystic, or mixed lesions according to the size and architecture of the cystic spaces, a distinction that is clinically relevant because it influences both treatment selection and expected response [2,3]. The head and neck region is one of the most frequently involved anatomical sites, where these lesions may cause aesthetic deformity, compressive symptoms, recurrent swelling, infection, or functional impairment depending on their extension and anatomical relationships [2,3].

Management of head and neck LMs remains challenging and must be individualized. Available treatment options include observation in selected asymptomatic cases, surgical excision, laser therapy, image-guided sclerotherapy, or combined strategies [3,4]. In current practice, percutaneous sclerotherapy is widely used, especially for macrocystic lesions, because it is less invasive than surgery and can achieve substantial volume reduction while limiting morbidity in anatomically complex regions [4]. Systematic review data support sclerotherapy as an effective treatment modality for head and neck LMs, although outcomes vary according to lesion subtype and the sclerosant used [4].

Among available sclerosants, doxycycline has shown favourable efficacy and safety in head and neck LMs, particularly in predominantly macrocystic lesions [5,6,7]. Ultrasound guidance is central to these procedures because it allows lesion targeting, aspiration, and drug delivery in real time, yet most reported series rely on conventional linear or curvilinear probes, and the specific contribution of higher-frequency superficial imaging, in terms of needle-tip control, delineation of superficial locules, and real-time appreciation of sclerosant distribution, has not been described in detail. Compared with conventional probes, higher-frequency transducers provide finer axial and lateral resolution of superficial structures, although at the expense of reduced depth of penetration for deeper components [6,7,8]. In superficial vascular anomalies, high-frequency linear transducers may be particularly useful because they improve spatial resolution of the skin, subcutaneous tissues, superficial cystic spaces, and septations, although at the cost of reduced penetration depth [9,10,11,12]. In this context, the present report focuses not on doxycycline sclerotherapy as a novel treatment, but on the procedural value of high-frequency ultrasound guidance with a small-footprint hockey-stick probe in a superficial submandibular macrocystic LM.

Here, we present the case of a submandibular macrocystic LM successfully managed with intralesional doxycycline under high-frequency ultrasound guidance, emphasizing the technical aspects of this imaging approach for superficial compartment identification and real-time monitoring of sclerosant distribution.

## 2. Case Presentation

A 32-year-old man with no relevant medical history was referred to our department for evaluation of a left submandibular swelling that had first been noticed approximately two years earlier and had slowly persisted over time. The patient reported no history of local infection, fever, pain, tenderness, dysphagia, dyspnoea, or other symptoms suggestive of upper aerodigestive compression. He did, however, report occasional spontaneous reduction in the swelling after exposure to heat. The lesion had become aesthetically bothersome over time, to the extent that the patient habitually grew a beard to conceal the cervical asymmetry. On inspection, the lesion occupied the entire left submandibular region and extended posteriorly toward the ipsilateral retromandibular angle region, with an estimated maximum clinical diameter of approximately 6.5 cm. On palpation, it was soft, fluctuant, painless, and easily deformable. Its mobility over the deeper planes was difficult to assess because of its marked softness and fluctuant nature. The overlying skin was intact, with no inflammatory changes, discoloration, ulceration, or evidence of cutaneous infiltration. No clinically evident compressive symptoms or associated neurological deficits were present.

Initial ultrasound examination showed, in the left submandibular region anteromedial to the ipsilateral submandibular gland, a multiloculated lesion with well-defined margins and finely corpusculated anechoic fluid content, measuring approximately 7.6 × 2.5 × 2.25 cm. Because of the lesion site and its sonographic relationship to the submandibular gland, a salivary origin was initially considered in the differential diagnosis. For this reason, fine-needle aspiration cytology (FNAC) was performed and interpreted according to the Milan System for Reporting Salivary Gland Cytopathology [13]. The first cytological examination was classified as diagnostic category III, namely atypia of undetermined significance, and described a “cystic lesion containing mucin”. The differential diagnosis included a benign mucinous cyst, but a low-grade mucoepidermoid carcinoma could not be excluded. Correlation with the clinical and radiological findings, as well as follow-up and possible repeat sampling or surgical excision, was therefore recommended. A second FNAC, performed six months later, yielded essentially overlapping findings, again describing a cystic lesion containing mucin and again categorized as atypia of undetermined significance. The report emphasized that the differential diagnosis could include lesions with different biological behaviour, ranging from benign mucous retention cysts to low-grade mucoepidermoid carcinoma, and concluded that definitive diagnosis required histological examination. These repeated indeterminate cytological results, together with the lesion site and the persistent uncertainty regarding its exact nature, prompted further tissue assessment. Magnetic resonance imaging demonstrated a well-encapsulated lesion in the left submandibular region measuring up to 56 × 35 mm, with well-defined margins and homogeneous fluid-signal content. The lesion extended into the internal paralaryngeal space and caused mild compression of the laryngeal wall, without radiological evidence of an obvious solid component on the imaging available or evaluation (Figure 1).

The combination of the lesion’s cystic morphology, deep extension, and non-conclusive cytological sampling supported the need for histological clarification before any definitive therapeutic strategy was selected.

The patient was therefore admitted for surgical exploration and biopsy under ordinary inpatient care. Preoperatively, informed consent was obtained, and the patient was made aware that the surgical strategy and the extent of any resection might be modified intraoperatively according to the frozen-section findings. The aim of this stepwise approach was to exclude malignancy before proceeding to a more conservative or lesion-directed treatment.

Intraoperative frozen-section examination showed fibrous stromal tissue containing multiple vascular structures and a mild chronic inflammatory infiltrate. Within this stroma, several cystically dilated spaces lined by cells without significant cytological atypia were observed; however, the lesion could not be further characterized with certainty on frozen section alone, and definitive diagnosis was deferred to permanent histology. Considering the absence of clear evidence of epithelial malignancy on intraoperative evaluation, the procedure was limited to an incisional biopsy rather than extended resection. A Redon suction drain was placed, and the patient had an uneventful postoperative course. He was discharged after 48 h, with no swelling, oedema, burning sensation, wound complications, or other postoperative adverse events.

Definitive histopathological examination of the incisional biopsy specimen, together with review of the frozen-section material, showed partially myxoid fibrous stroma composed of spindle-shaped fibroblastic-myofibroblastic cells, positive for smooth muscle actin and calponin. Within this stroma, cystically dilated spaces lined by endothelial cells of probable lymphatic nature were identified. These cells were positive for CD34, CD31, and podoplanin. The cystic spaces contained amorphous proteinaceous material and scattered histiocytes. No epithelial structures were observed, with negativity for CKAE1/AE3, p40, and EMA. Overall, the morphological and immunohistochemical findings were consistent with a lymphatic vascular proliferation, with no evidence of epithelial neoplasia, thereby supporting the diagnosis of macrocystic lymphatic malformation.

After malignant disease had been excluded and a lymphatic malformation had been histologically confirmed, treatment options were reconsidered according to the lesion subtype, location, and clinical presentation. Given the macrocystic nature of the lesion, the absence of acute symptoms, the cervical site, and the possibility of avoiding a more invasive surgical excision in an anatomically complex region, a minimally invasive approach with intralesional doxycycline was selected. Sclerotherapy was considered particularly appropriate in this setting because it offered the possibility of lesion reduction with minimal morbidity while preserving surrounding structures.

The first sclerotherapy session was scheduled approximately six weeks after the biopsy procedure to allow adequate healing of the operated region. The treatment was performed in a day-hospital setting with same-day discharge. After topical local anesthesia with 1% lidocaine gel, the patient was positioned supine with neck hyperextension. A linear hockey-stick transducer with a frequency range of 6–18 MHz was applied over the lesion. Ultrasound confirmed the presence of a multiloculated cystic mass located anteromedially to the ipsilateral submandibular gland, characterized by well-defined margins and finely corpusculated anechoic-fluid content. The use of a high-frequency probe provided detailed visualization of the superficial soft-tissue planes, including skin and subcutaneous tissues, and allowed accurate identification of the superficial aspects of the lesion and of the main target locule. Under continuous ultrasound guidance, a 25-gauge needle was advanced into the largest cystic cavity using a single puncture in-plane approach. Aspiration was first performed to decompress the lesion and confirm correct intralesional needle placement. Subsequently, doxycycline was prepared at a concentration of 10 mg/mL in normal saline, and a total dose of 100 mg was slowly injected into the lesion under continuous sonographic monitoring. A single-puncture strategy was adopted, targeting the dominant macrocystic locule, which represented the largest fluid compartment and appeared to communicate with the adjacent smaller locules on real-time imaging; decompression and refilling of these neighbouring compartments during aspiration and injection suggested at least partial intercommunication, so that a single access point was considered sufficient rather than performing multiple separate punctures. The needle was inserted with a shallow in-plane trajectory, advancing the shaft parallel to the long axis of the transducer so that the full needle and its tip remained continuously visualized, and was directed from a lateral cervical entry point toward the centre of the dominant locule while keeping the trajectory superficial to the deeper paralaryngeal extension. During advancement, care was taken to keep the needle path away from the ipsilateral submandibular gland, the major cervical vessels, and the deeper laryngeal wall, all of which were identified sonographically before puncture. Approximately 20–25 mL of finely corpusculated fluid was aspirated from the dominant locule before sclerosant instillation. Real-time monitoring during injection was based on predefined objective sonographic criteria: continuous visualization of the needle tip within the target cavity, progressive echogenic filling and expansion of the locule by the injected solution, homogeneous distribution of the sclerosant within the compartment, and absence of leakage or extravasation into the surrounding soft tissues. The 10 mg/mL concentration was selected because it represents the most reported doxycycline concentration for lymphatic malformation sclerotherapy, particularly in pediatric head and neck series and systematic reviews, whereas concentrations up to 20 mg/mL have been described as upper reported concentrations [14]. This concentration also allows predictable preparation of a 100 mg/10 mL solution and real-time volume-controlled intralesional injection. The decision to inject 10 mL, despite an estimated cystic volume of approximately 25–35 mL, was based on a conservative volume-guided strategy: after decompression of the dominant locule, the sclerosant volume was tailored to the superficial cervical location, sonographic refilling of the cavity, and avoidance of tension within the lesion. This approach is consistent with published sclerotherapy techniques in which cystic contents are aspirated before sclerosant instillation and the injected volume is adjusted according to the aspirated volume, cyst capacity, and procedural safety considerations [14,15]. The total dose of 100 mg therefore reflected both standard single-vial preparation and a conservative dose-volume choice for a superficial cervical target. Real-time imaging allowed direct observation of sclerosant distribution within the lesion and immediate confirmation that the injected solution remained confined to the target compartment (Figure 2).

At the end of the procedure, the needle was withdrawn and a compressive dressing was applied.

The patient was discharged later the same day. No intraoperative pain, immediate adverse effects, or post-procedural complications were reported. Post-treatment management consisted only of non-steroidal anti-inflammatory drugs as needed.

At the first clinical follow-up, the lesion showed a partial reduction in volume, indicating a favourable but incomplete response. This reduction was assessed clinically, on the basis of the appreciable decrease in size and prominence of the palpable swelling relative to baseline; objective volumetric or dimensional measurements and post-treatment imaging were not obtained at this time point, so the magnitude of the reduction could not be quantified. Because residual tumefaction was still clinically appreciable, a second session of ultrasound-guided doxycycline sclerotherapy was planned. This second treatment was performed three months after the first one, following the same protocol, again in a day-hospital setting with morning admission and discharge later the same day. The procedure was again well tolerated, with no complications or clinically relevant side effects.

At one-year follow-up, the patient reported apparent complete clinical resolution of the cervical swelling and full satisfaction with the aesthetic result (Figure 3). Clinical photographs documented normalization of the cervical profile compared with the pre-treatment condition. No post-treatment ultrasound or MRI was obtained because the patient had moved abroad and, in the setting of complete symptomatic and clinical improvement, declined further radiological investigations. Resolution was therefore assessed on clinical and photographic grounds only, and the response of the deeper paralaryngeal component of the lesion could not be confirmed radiologically; the possibility of residual subclinical disease in this region cannot be excluded, and the clinical photographs document the external cervical profile but do not constitute radiological confirmation of resolution. Written informed consent for publication of clinical data and images was obtained from the patient.

## 3. Discussion

Lymphatic malformations of the head and neck represent a heterogeneous group of low-flow vascular anomalies that may vary considerably in size, depth, compartmentalization, and clinical behaviour [1,2,3]. Although classically regarded as congenital lesions, they may become clinically evident later in life or attract medical attention only when their volume increases, cosmetic asymmetry becomes more pronounced, or they produce symptoms due to secondary infection, haemorrhage, or compression of adjacent structures [2,3]. In adults, the differential diagnosis of a lateral or submandibular cystic mass is often broad and may include salivary lesions, branchial cleft cysts, ranulas, cystic metastatic disease, and other benign or malignant cervical masses. In this context, a macrocystic LM may not be the first diagnostic consideration, particularly when the lesion appears to be closely related to the submandibular gland.

This diagnostic issue was clearly illustrated in the present case. Because of the lesion’s location and the initial ultrasound findings, a salivary origin was initially suspected, and cytological evaluation was interpreted within the Milan System for Reporting Salivary Gland Cytopathology [13]. In both samplings, the lesion yielded a mucin-containing cystic specimen categorized as atypia of undetermined significance. These findings did not establish a definitive diagnosis and, importantly, did not completely exclude a low-grade mucoepidermoid carcinoma. As a result, the patient pathway could not proceed directly to sclerotherapy. Histological confirmation first became necessary to rule out malignancy and avoid an inappropriate minimally invasive treatment of a potentially neoplastic lesion. Before incisional biopsy was undertaken, the less invasive diagnostic options had effectively been exhausted: two separate fine-needle aspiration cytology samples obtained six months apart had both returned an indeterminate, atypia-of-undetermined-significance result that could not distinguish a benign mucinous cyst from a low-grade mucoepidermoid carcinoma, while magnetic resonance imaging confirmed a cystic lesion but could not characterize its biological nature. In this setting, repeating FNAC was unlikely to resolve the uncertainty; the predominantly fluid-filled cystic architecture made core-needle biopsy liable to sampling error, and simple observation was inappropriate given the unexcluded possibility of malignancy. An incisional biopsy with intraoperative frozen-section assessment was therefore selected as the lowest-risk option able to provide a definitive tissue diagnosis while allowing the procedure to remain conservative once malignancy had been excluded. This step was considered essential precisely because the submandibular location, the relationship to the salivary gland, and the mucin-containing cytology raised a realistic concern for a low-grade salivary malignancy that had to be ruled out before any sclerosing agent was injected. From a clinical standpoint, this sequence represents one of the key learning points of the case: although imaging and aspiration findings may suggest a benign cystic process, persistent diagnostic uncertainty in a submandibular lesion justifies tissue diagnosis before selecting a definitive treatment strategy.

The final histological and immunohistochemical diagnosis of lymphatic vascular proliferation without epithelial neoplasia was crucial in guiding treatment selection. In this patient, the therapeutic decision was driven less by general management principles than by three case-specific factors: the macrocystic architecture of the lesion, its superficial but anatomically confined submandibular and paralaryngeal location, and the relevant aesthetic burden despite the absence of urgent compressive symptoms. Surgical excision could have provided definitive removal, but it would have required dissection in a compact cervical region close to the submandibular gland, adjacent soft tissues, and upper aerodigestive structures. Laser therapy was not considered appropriate because the lesion was not a superficial mucosal microcystic malformation. Therefore, repeatable image-guided sclerotherapy represented a proportionate strategy, offering the possibility of lesion reduction while limiting access-related morbidity [3,4].

The choice of doxycycline should also be interpreted in the broader context of available sclerosants. In the systematic review and meta-analysis by De Maria et al., including 25 studies and 726 patients with head, face, and neck LMs, the overall complete cure rate after percutaneous sclerotherapy was 50.5%, while macrocystic lesions showed a higher cure rate than microcystic and mixed lesions. In that analysis, doxycycline showed the highest reported complete cure rate among the evaluated agents, although the authors emphasized heterogeneity across studies and lesion subtypes [4]. Earlier reports, including the study by Cordes et al., supported the use of doxycycline as a primary treatment for head and neck LMs [5]. Jamal et al. further described encouraging outcomes in paediatric head and neck macrocystic lesions treated with doxycycline, emphasizing its practicality and tolerability [6]. More recently, Maleux et al. reported an intermediate series of 27 cases confirming the usefulness of doxycycline sclerotherapy for head and neck LMs [7]. These data support the use of doxycycline in a predominantly macrocystic lesion such as the present one, while also emphasizing that incomplete response after a single session remains compatible with the expected behaviour of LMs, especially when multiloculated.

Other sclerosants may be appropriate in selected settings. OK-432/Picibanil has been widely used for macrocystic LMs and, in a direct comparison with doxycycline, showed similar clinical success rates [15]. However, OK-432 required a higher number of injections and was associated with more early postoperative swelling [15]. Sodium tetradecyl sulfate has also been used in paediatric head and neck LMs, either alone or in comparison with doxycycline, with reported safety and efficacy but without standardized protocols [16]. Polidocanol microfoam has shown promising results in head and neck venous and lymphatic malformations, with particularly favourable volume reduction in macrocystic LMs, although the available evidence remains limited [17]. Bleomycin and ethanol are also included among sclerosants used for low-flow vascular malformations, but their safety profile and indications differ from those of doxycycline. Ethanol is generally approached cautiously in superficial cervical lesions because of pain, tissue injury, nerve damage, and systemic adverse effects reported in the broader low-flow vascular malformation literature [18,19].

Recent radiology series support the role of image-guided sclerotherapy as a safe and reproducible treatment strategy for lymphatic malformations. Bouwman et al. conducted a review on 116 children treated with 234 sclerotherapy procedures for lymphatic or venolymphatic malformations and reported that all patients achieved some or a good clinical response, even though mixed macrocystic and microcystic lesions required more than one procedure and were more difficult to treat effectively [20]. Similarly, Yamaki et al. reported a 13-year experience with percutaneous ultrasound-guided sclerotherapy using 3% polidocanol microfoam in 32 patients with lymphatic malformations, nearly half of which involved the head and neck region. Excellent or moderate responses were observed in 88% of patients, with particularly favourable results in macrocystic lesions and a low rate of inflammatory post-treatment symptoms [20].

However, the main scientific interest of the present report does not lie in the use of doxycycline per se, which is already supported by the literature [5,6,7], but rather in the procedural use of an 18 MHz small-footprint hockey-stick probe for a superficial submandibular LM. In this lesion, high-frequency ultrasound appeared to provide high spatial resolution and clear visualization of the superficial soft-tissue planes, with identification of the dominant cystic compartment, in-plane needle-tracking and real-time assessment of sclerosant distribution.

This point is particularly relevant because ultrasound guidance in sclerotherapy is not limited to puncture confirmation. In a multiloculated lesion, the operator must also understand whether the sclerosant is spreading within the intended compartment, whether refilling produces excessive tension, and whether adjacent superficial structures remain safely separated from the treated cavity. The use of a high-frequency hockey-stick probe facilitated these steps in the present case. Nevertheless, this observation should be interpreted cautiously: the report does not demonstrate superiority over standard linear probes but suggests that high-frequency superficial imaging may improve procedural confidence and technical control in selected superficial cervical LMs [9,10,11,12].

An additional practical lesson from this case concerns the dose-volume strategy. The first session used 10 mL of doxycycline solution despite an estimated cystic volume of approximately 25–35 mL. This was intentionally conservative because the lesion was superficial, cervical, and close to functionally relevant structures; excessive refilling of the cavity after aspiration could have increased intralesional tension, discomfort, leakage, or local inflammatory swelling. However, this same conservative strategy may also have contributed to the incomplete response after the first session. In a multiloculated LM, a limited injected volume may not expose all residual locules and septate compartments to an adequate concentration-time effect of the sclerosant. Therefore, the need for a second session was plausibly related not only to the intrinsic multiloculated architecture of the malformation, but also to the intentionally limited volume treated during the first procedure. This interpretation does not indicate technical failure; rather, it supports the rationale for staged, volume-controlled sclerotherapy in selected superficial cervical lesions, where safety and avoidance of excessive local pressure may be prioritized over single-session completeness.

Both injections were well tolerated and required only topical local anesthesia, same-day hospitalization, and simple postoperative management with non-steroidal anti-inflammatory medication. This favourable perioperative course is clinically relevant because it supports the feasibility of repeating the procedure when the first treatment produces only a partial response, which is not uncommon in macrocystic or multiloculated lesions [4,5,6,7].

This report has several limitations. First, the outcome was assessed clinically, and no post-treatment ultrasound or MRI was available because the patient moved abroad and declined further imaging after complete symptomatic and aesthetic resolution. As a result, objective volumetric reduction, residual subclinical locules, and radiological recurrence could not be quantified. Second, the relationship between the conservative injected volume and the incomplete response after the first session can only be considered plausible, not proven. The first injection volume was deliberately limited for safety reasons in a superficial cervical target, but this may have reduced sclerosant exposure of residual locules and contributed to the need for retreatment. Third, the report does not isolate the effect of high-frequency ultrasound from the effect of doxycycline itself, nor does it compare the hockey-stick probe with conventional linear probes. Finally, as a single-case report, the findings cannot be generalized to all head and neck LMs, particularly deeper, microcystic, mixed, or syndromic lesions.

Nevertheless, the clinical outcome remained meaningful. The lesion had originally produced a visible submandibular deformity affecting patient’s self-presentation, but at one year the swelling had resolved clinically, and the patient reported full satisfaction with the aesthetic result. The absence of radiological follow-up should therefore be acknowledged, but it does not negate the clinical relevance of the observed response.

From a clinical perspective, the main lessons learned are threefold. First, persistent diagnostic uncertainty in a cystic submandibular mass may justify histological confirmation before minimally invasive treatment is undertaken. Second, doxycycline sclerotherapy remains a valid and low-morbidity option for selected macrocystic head and neck LMs [5,6,7]. Third, in superficial cervical lesions, high-frequency ultrasound may serve not simply as a guidance modality, but as a technical enhancement that improves procedural precision and operator confidence [9,10,11,12,21].

## 4. Conclusions

This case report illustrates the feasible use of high-frequency ultrasound as a technical adjunct during doxycycline sclerotherapy for a superficial macrocystic head and neck lymphatic malformation. In our experience, the 18 MHz hockey-stick probe facilitated needle placement, visualization of superficial cystic compartments, and real-time monitoring of sclerosant distribution during treatment. The case is also noteworthy from a diagnostic perspective, as repeated FNAC results were inconclusive, and histological confirmation was required before appropriate minimally invasive treatment could be safely selected.

Although doxycycline sclerotherapy is already established for selected macrocystic LMs, this case suggests that high-frequency superficial sonography may represent a useful procedural refinement in anatomically confined superficial regions such as the submandibular area. The partial response after the first session also emphasizes that conservative volume-controlled injection may require staged retreatment, particularly in multiloculated lesions. Larger case series with standardized imaging follow-up are needed to determine whether this technical approach improves visualization, reproducibility and documentation of sclerosant distribution, since it is not possible to infer clinical superiority from a single case.

## Figures and Tables

**Figure 1:**
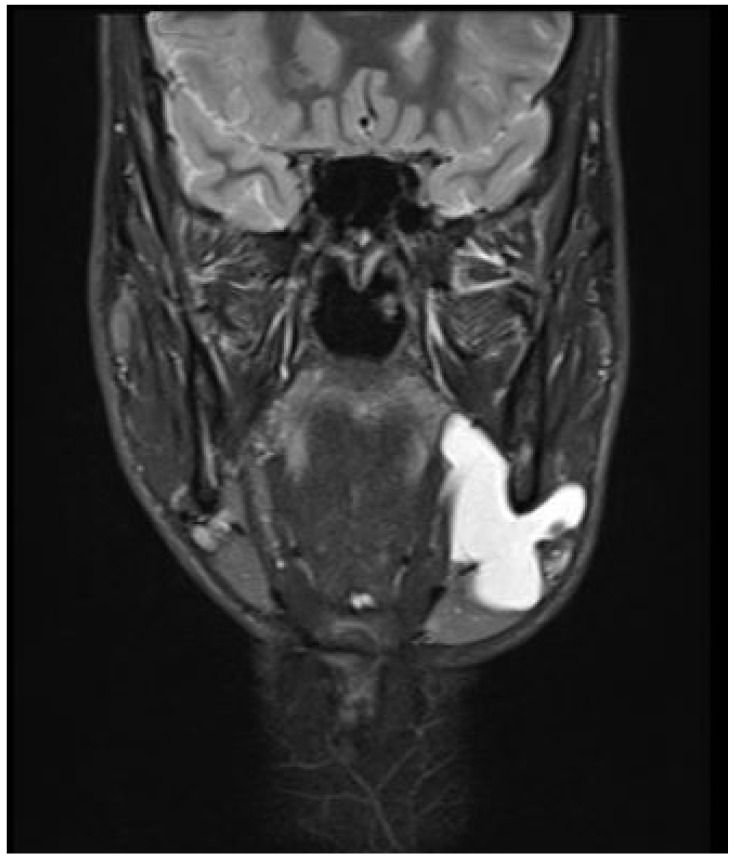
Preoperative MRI showing the presence of a well-encapsulated lesion in the left submandibular region with homogeneous fluid-signal content.

**Figure 2:**
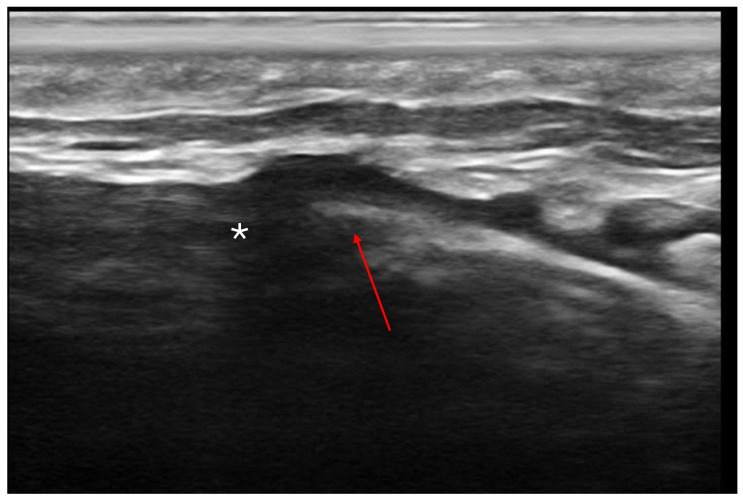
Intraprocedural high-frequency ultrasound image (18 MHz hockey-stick transducer) acquired during intralesional doxycycline injection using an in-plane approach. The arrow indicates the 25-gauge needle, with its tip positioned within the target cystic cavity; the asterisk (*) marks the lumen of the dominant locule. The high-frequency probe allows visualization of the superficial soft-tissue planes overlying the lesion and of the needle trajectory within the cyst. Arrows and labels have been added to the original image to indicate the needle, the needle tip, the lesion lumen, and the superficial soft-tissue planes.

**Figure 3:**
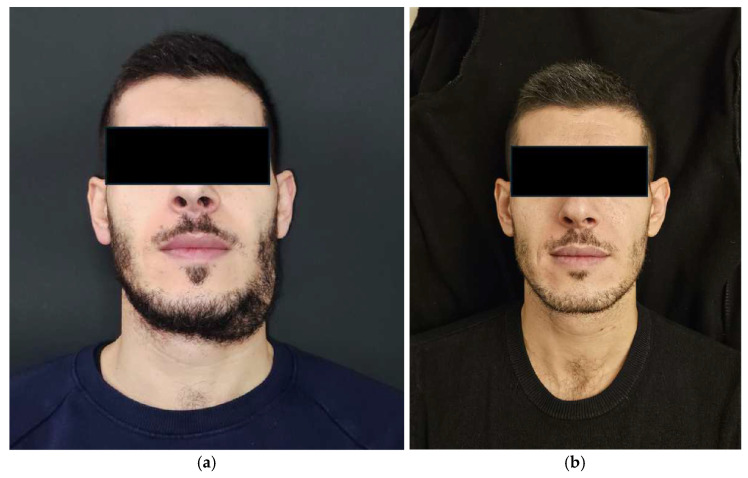
Clinical images. (**a**) Before the treatment: in the left submandibular region, a well-visible swelling is present. (**b**) 1-Year post-treatment follow-up: the swelling is completely absent.

## Data Availability

This manuscript is a single-patient case report, and therefore no tabular dataset was generated or analysed; the clinical information supporting the findings is contained within the article itself. To support the imaging findings, we have deposited the anonymized ultrasound clips and image used in the study in a dedicated repository, available at: https://github.com/faustofiori/Healthcare_Ultrasound (accessed on 12 March 2026). All identifying information (patient name, identifiers, institution, and examination date) has been removed from the files, and only the diagnostic ultrasound content has been retained. The individual clinical data cannot be shared beyond this anonymized material for privacy and confidentiality reasons, in accordance with the patient’s written informed consent and applicable data-protection regulations.

## Abbreviations

The following abbreviations are used in this manuscript:

LM	Lymphatic malformations
MRI	Magnetic resonance imaging
FNAC	Fine-needle aspiration cytology
